# Genetic Liability to Opioid Use Disorder and Dementia‐Related Biological Pathways: A Mendelian Randomisation Study

**DOI:** 10.1002/gps.70270

**Published:** 2026-11-06

**Authors:** Sara Javidnia, Héléne Toinét Cronjé, Ville Karhunen, Dipender Gill, Steven Bell, Joseph D. Deak, Daniel Levey, Rachel L. Kember, Henry R. Kranzler, Stephen Burgess, Joel Gelernter, Klaus P. Ebmeier, Anya Topiwala

**Affiliations:** ^1^ Department of Psychiatry and Oxford Centre for Integrative Neuroimaging University of Oxford Oxford UK; ^2^ MRC Biostatistics Unit University of Cambridge Cambridge UK; ^3^ Faculty of Medicine Research Unit of Population Health University of Oulu Oulu Finland; ^4^ Department of Epidemiology and Biostatistics School of Public Health Imperial College London London UK; ^5^ Department of Behavioural Science and Health Epidemiology of Cancer Healthcare & Outcomes (ECHO) Group University College London London UK; ^6^ Department of Epidemiology and Public Health University College London London UK; ^7^ Department of Psychiatry Yale University School of Medicine New Haven Connecticut USA; ^8^ Veterans Affairs Connecticut Healthcare System West Haven Connecticut USA; ^9^ Department of Psychiatry University of Pennsylvania Perelman School of Medicine Philadelphia Pennsylvania USA; ^10^ Mental Illness Research Education and Clinical Center Crescenz Veterans Affairs Medical Center Philadelphia Pennsylvania USA; ^11^ Department of Public Health and Primary Care School of Clinical Medicine University of Cambridge Cambridge UK; ^12^ Nuffield Department of Population Health Big Data Institute University of Oxford Oxford UK

**Keywords:** C‐reactive protein, dementia, inflammation, mendelian randomisation, neurodegeneration, opioid use disorder

## Abstract

**Objectives:**

Opioid use disorder (OUD) is increasingly recognised as a risk factor for cognitive impairment and dementia, but the biological mechanisms underlying this association remain unclear. We aimed to investigate the involvement of established neurodegenerative, vascular, or inflammatory pathways.

**Methods:**

We performed a two‐sample Mendelian randomisation study using summary‐level genetic data from nine genome‐wide association studies of individuals predominantly of European ancestry. Genetic instruments for OUD liability were used to estimate associations with neurodegenerative, vascular, and inflammatory phenotypes, including amyloid‐β, tau, cerebral amyloid angiopathy, cerebral microinfarcts, and C‐reactive protein (CRP). Causal effects were estimated using inverse‐variance weighted Mendelian randomisation, with replication and Bayesian colocalisation analyses used to further evaluate the findings.

**Results:**

There was little evidence that genetic liability to OUD was associated with amyloid‐β, tau, or vascular phenotypes. A modest positive association was observed with CRP (*β* = 0.11, 95% CI 0.04–0.18, *p* = 2 × 10^−3^). This association was replicated in an independent dataset (*β* = 0.12, 95% CI 0.05–0.19, *p* = 1.59 × 10^−3^). A nominal inverse association was also observed with IL‐15 (*β* = −0.23, 95% CI −0.43 to −0.03, *p* = 2.2 × 10^−2^), although colocalisation did not support a shared causal variant at implicated loci.

**Conclusions:**

Genetic liability to OUD showed little evidence of association with the neurodegenerative or vascular phenotypes examined; however, these findings do not exclude the involvement of Alzheimer's disease or cerebrovascular pathways, particularly given the limited power and the use of only three genetic instruments. The observed inflammatory associations should be considered hypothesis‐generating, particularly given the lack of colocalisation support, and require further investigation.

## Key Points

- Genetic liability to opioid use disorder showed a modest association with higher circulating C‐reactive protein levels.
- No evidence was found for associations with major neurodegenerative or vascular biomarkers linked to dementia, although these findings do not exclude Alzheimer's disease or cerebrovascular pathways, particularly given limited power.
- The inflammatory findings are hypothesis‐generating and should be interpreted cautiously given the use of only three genetic instruments and the lack of colocalisation support.

## 1 Introduction

Opioid use disorder (OUD) is an escalating public health concern with significant implications for brain health. Individuals with OUD have substantially elevated risk of cognitive impairment and dementia, and recent Mendelian randomisation (MR) analyses support a potential causal relationship with all‐cause dementia [1, 2]. However, the biological mechanisms underlying this association remain unclear, limiting opportunities for targeted prevention and intervention.

Chronic opioid exposure has been associated with structural brain changes, including reductions in hippocampal and white matter volumes, alongside decline in fluid intelligence and executive functioning [3]. Opioids may contribute to neurodegeneration through direct receptor‐mediated effects, and indirectly via co‐occurring factors such as chronic pain, psychological stress, and altered neuroinflammatory signalling [4]. However, it remains unclear whether these changes represent causal pathways to dementia or downstream consequences of OUD and its comorbidities.

There is limited evidence on whether OUD influences established neuropathological processes underlying dementia, including amyloid beta and tau pathology, or vascular mechanisms, such as small vessel disease. The potential role of systemic inflammation, which has been implicated in both OUD and dementia [5], also remains uncertain.

To address this gap, we used two‐sample Mendelian randomisation and Bayesian colocalisation to investigate whether genetic liability to OUD is associated with dementia‐related neurodegenerative, vascular, and inflammatory traits, including amyloid beta, tau, cerebral amyloid angiopathy, microinfarcts, and C‐reactive protein (CRP) [6, 7, 8, 9, 10]. By evaluating these pathways, we sought to determine whether human genetic evidence supports a role for OUD in increasing dementia risk through its potential effects on established dementia‐related mechanisms.

## 2 Materials and Methods

### 2.1 Sample and Data Sources

We used publicly available summary statistics from genome‐wide association studies (GWASs) conducted in predominantly European ancestry populations. Sample sizes varied across the individual exposure and outcome GWASs and are reported in Supporting Information S2: Tables 1 and 2.

### 2.2 Opioid Use Disorder Trait

Genetic variants associated with OUD were obtained from a large GWAS meta‐analysis comprising 7 cohorts (*n* = 15,251 cases, *n* = 538,935 controls) [11] (Supporting Information S2: Table 2). Lifetime OUD was identified from International Classification of Diseases (ICD‐9/10) codes in linked electronic health records (EHR) (Supporting Information S2: Table 3). Individuals without an OUD diagnosis were classified as controls [1, 11]. Three independent single‐nucleotide polymorphisms (SNPs) (*r* ^2^ < 0.1) reached genome‐wide significance (*p* < 5 × 10^−8^) and were used as instrumental variables in the MR analysis: rs79704991 and rs1799971, located in the *OPRM1* gene, and rs11372849, located in the *FURIN* gene (Supporting Information S2: Table 4). Where rs11372849 was not available in the outcome dataset, a proxy SNP in high linkage disequilibrium (rs17514846; *r* ^2^ > 0.8) was used instead [1]. Pairwise linkage disequilibrium between the genetic instruments was assessed. The two *OPRM1* variants (rs79704991 and rs1799971) showed low LD (*r* ^2^ = 0.02), while rs11372849 in *FURIN* is located on a different chromosome. Given the low correlation between the *OPRM1* instruments, all variants were treated as independent in the IVW analyses; residual LD was not incorporated into the IVW variance calculation.

### 2.3 Neurobiological and Inflammatory Biomarkers

Genetic associations for neuroendophenotypes and circulating inflammatory and neurodegeneration‐related biomarkers were obtained from previously published GWAS studies. These included 11 neuropathological traits (*n* = 7804) [6] (Supporting Information S2: Table 1); plasma tau levels (*n* = 14,721) [8]; tau and amyloid‐beta levels in cerebrospinal fluid (CSF) (*n* = 13,116) [7]; plasma neurofilament light chain (NfL) levels measured using bead‐based immunoassays (*n* = 18,532) and antibody‐based Olink proteomic measurements (*n* = 49,682) [12, 13]; and Olink‐derived measures of circulating protein abundance in UK Biobank participants (*n* ≤ 54,219) [13], including Glial Fibrillary Acidic Protein (GFAP), Amyloid Precursor Protein (APP), Brain‐Derived Neurotrophic Factor (BDNF), Microtubule‐Associated Protein Tau (MAPT), interleukin‐4 (IL‐4), and interleukin‐15 (IL‐15) [13]. Additional inflammatory biomarkers included interleukin‐1α (IL‐1α; *n* = 47,745) [14], interleukin‐6 (IL‐6; *n* = 74,679), interleukin‐1β (IL‐1β; *n* = 38,868), interleukin‐18 (IL‐18; *n* = 70,126), and (Monocyte Chemoattractant Protein‐1, MCP‐1; *n* = 74,783) [15]; as well as C‐reactive protein (CRP) levels from primary and replication GWAS datasets (*n* = 575,531 and *n* = 319,548, respectively) [9, 10]. The scale of the MR estimates was determined by the scale of the SNP–exposure and SNP–outcome associations in the respective GWASs. OUD was analysed as a binary exposure, with SNP–OUD associations expressed on the log‐odds scale. Outcomes comprised continuous, ordinal, and binary phenotypes, with measurement scales, transformations, and standardisation varying across GWASs. Accordingly, MR estimates were interpreted on the scale of the respective outcome per unit increase in the log‐odds of genetic liability to OUD. The phenotype type, measurement or transformation, standardisation, and scale of the SNP–outcome associations for each outcome are detailed in Supporting Information S2: Table 1.

Potential sample overlap between the OUD exposure GWAS and each outcome GWAS was assessed based on the contributing cohorts reported in the source studies. Potential overlap was identified for the CRP replication analysis, as FinnGen contributed to both the OUD GWAS and the CRP dataset [10]. The exact number of overlapping participants could not be quantified from the publicly available summary‐level data. Potential cohort overlap for all outcome GWASs is reported in Supporting Information S2: Table 1.

### 2.4 Genetic Analysis

Two‐sample MR was employed to evaluate the association between genetic liability to OUD and neuroendophenotype or inflammatory cytokine outcomes. The inverse variance weighted (IVW) method was used as the primary analytical approach. Analyses were conducted using the *TwoSampleMR* (version 0.6.3) and *MendelianRandomization* packages in R. Additional sensitivity analyses, including Cochran's *Q* tests for heterogeneity and leave‐one‐out analyses, were conducted using *TwoSampleMR* version 0.6.22. Mendelian randomisation analyses were conducted under the three‐core instrumental variable assumptions. First, the relevance assumption requires that the genetic instruments are robustly associated with the exposure (OUD). Instruments were selected based on genome‐wide significant associations with OUD, and instrument strength was assessed using the proportion of variance explained (*R* ^2^) and F‐statistics, with *F* > 10 considered indicative of adequate instrument strength. Second, the independence assumption requires that the genetic instruments are independent of factors that confound the association between OUD and the outcomes. Third, the exclusion restriction assumption requires that the genetic instruments influence the outcomes only through their effect on OUD and not through alternative pathways (horizontal pleiotropy). Pairwise linkage disequilibrium between instruments was assessed to confirm their independence, and heterogeneity across SNP‐specific causal estimates was evaluated using Cochran's Q statistic. Leave‐one‐out sensitivity analyses were additionally performed by sequentially excluding each genetic instrument to assess whether the overall MR estimates were disproportionately influenced by any single variant. Leave‐one‐out analysis was not performed for IL‐1β because only two genetic instruments were available. Given the limited number of genetic instruments, the sensitivity analyses were interpreted cautiously. Heterogeneity and leave‐one‐out results are presented in Supporting Information S2: Tables 9 and 10, respectively. Replication and colocalisation analyses were undertaken to provide additional evidence supporting the robustness of the findings [16]. Prior to analysis, SNP‐exposure and SNP‐outcome associations were harmonised to ensure alignment on the effect allele. To assess potential confounding due to linkage disequilibrium, Bayesian colocalisation analysis was performed using the *coloc* package (version 5.2.3) [16]. Multiple testing correction was performed using false discovery rate (FDR) (Benjamini–Hochberg, 5%). The inflammatory biomarkers and neuroendophenotypes were treated as separate families of hypotheses because they represented distinct outcome domains addressing separate research questions. Benjamini–Hochberg correction was therefore applied separately across the eight inflammatory biomarkers and the 20 neuroendophenotypes, with the false discovery rate controlled at 5% within each family. Replication analyses were additionally performed for CRP using an independent GWAS dataset [10].

We used Bayesian colocalisation analysis to estimate posterior probabilities for five mutually exclusive hypotheses: no association with either trait (PP.H0), association with OUD liability only (PP.H1), association with the outcome only (PP.H2), association with both traits through distinct causal variants (PP.H3), and association with both traits through a shared causal variant (PP.H4) [17]. We first assessed evidence that both traits were associated within the locus using PP.H3 + PP.H4 > 0.5. Among loci meeting this criterion, support for a shared rather than distinct causal variant was summarised using PP.H4/(PP.H4 + PP.H3). Values > 0.5 were interpreted as greater posterior support for colocalisation than for distinct causal variants, whereas values < 0.5 were interpreted as greater support for distinct association signals. Colocalisation analyses were performed under the standard coloc assumption of a single causal variant per trait within each genomic region, and results were interpreted accordingly. This study was not preregistered.

## 3 Results

Three independent variants in *OPRM1* and *FURIN* associated with OUD at genome‐wide significance were selected as instrumental variables for MR analyses. Using the inverse‐variance weighted approach, greater genetic liability to OUD was associated with higher circulating CRP levels (inverse‐variance weighted [IVW] *β* = 0.11, 95% CI [0.04, 0.18], *p* = 2.21 × 10^−3^) (Figure 1) (Supporting Information S2: Table 5). Replication analyses using an independent CRP dataset demonstrated a consistent direction of effect and slightly stronger association estimates (IVW *β* = 0.12, 95% CI [0.05, 0.19], *p* = 1.59 × 10^−3^) (Supporting Information S2: Table 7). There was nominal evidence of an inverse association between genetic liability to OUD and circulating IL‐15 levels (IVW *β* = −0.23, 95% CI −0.43 to −0.03; *p* = 0.022), although this did not survive correction for multiple testing at the prespecified 5% FDR threshold (Supporting Information S2: Table 5).

**FIGURE 1:**
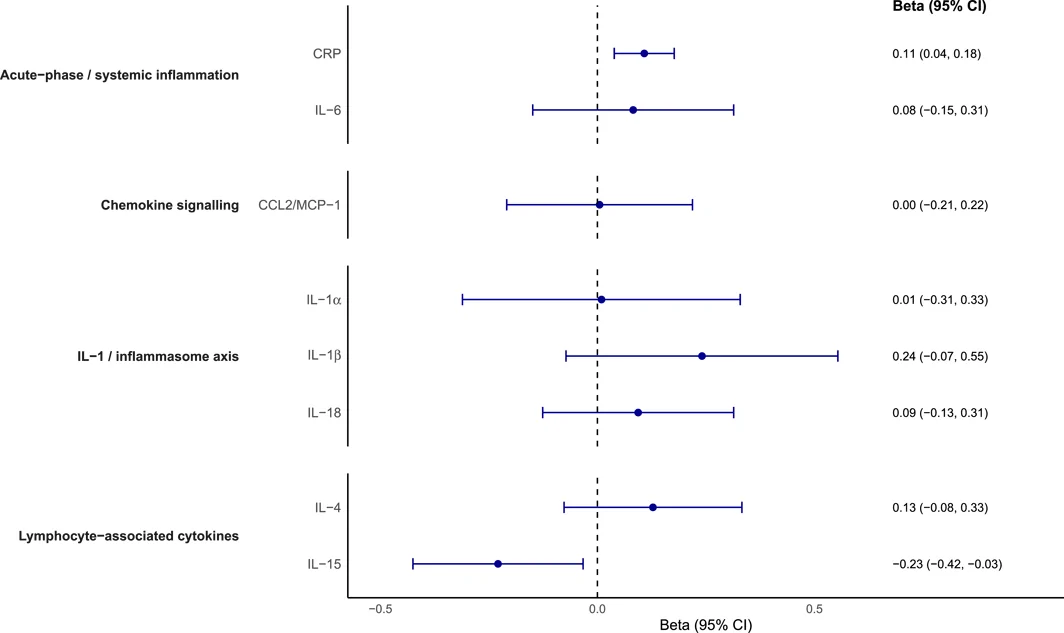
Two‐sample Mendelian randomisation estimates of the effect of genetic liability to opioid use disorder (OUD) on circulating inflammatory biomarkers. The plot displays beta coefficients and 95% confidence intervals for the effect of genetically predicted OUD on levels of selected inflammatory markers, including interleukins (IL‐6, IL‐4, IL‐1β, IL‐1α, IL‐18, IL‐15), C‐reactive protein (CRP), and MCP‐1(CCL2). Biomarkers are grouped by biological function. Source GWAS are detailed in Supporting Information S2: Tables 1 and 2. Estimates were derived using inverse‐variance weighted (IVW) MR. Beta coefficients represent the change in biomarker level per unit increase in the log‐odds of genetic liability to OUD.

For further details on the inflammatory biomarker analyses, see Supporting Information S1: Figure 3.

To assess whether this association reflected a shared underlying causal variant, we performed Bayesian colocalisation analyses at the genome‐wide significant loci. Colocalisation analyses did not support a shared association between OUD and CRP at either locus. For the primary CRP analysis, PP.H3 + PP.H4 was 0.196 at *OPRM1* and 0.040 at *FURIN*, below the predefined threshold of > 0.5. Corresponding values were lower in the replication analysis. Across these analyses, PP.H1, representing association with OUD alone, had the highest posterior probability (Figure 2). This suggests that the MR finding is unlikely to be explained by a single shared causal variant at these loci. Similar findings were observed for IL‐15 and the independent CRP replication dataset, with limited evidence supporting colocalisation across analyses Supporting Information S2: Table 8 and Supporting Information S1: Figures 1 and 2.

**FIGURE 2:**
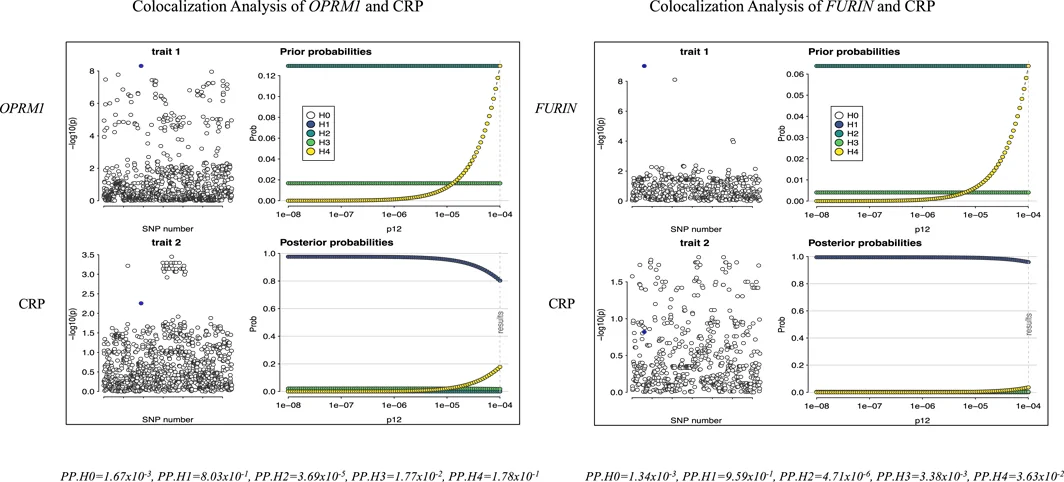
Colocalisation plots assess the overlap of association signals for opioid use disorder (OUD) and C‐reactive protein (CRP) at the *OPRM1* (left) and *FURIN* (right) loci. Each panel includes regional association plots for OUD (trait 1) and CRP (trait 2), as well as prior and posterior probabilities for hypotheses H0–H4. Analyses were conducted using the coloc package. Posterior probabilities (PP) for each hypothesis are shown below the plots.

Aside from the CRP association described above, no associations across the broader panel of inflammatory markers and neuroendophenotypes reached statistical significance after correction for multiple testing (Supporting Information S2: Tables 5 and 6; Figure 3). Whilst weak inverse associations between OUD liability and plasma neurofilament light chain levels (IVW *β* = −0.28, 95% CI [−0.49, −0.07], *p* = 9.99 × 10^−3^), and cerebral amyloid angiopathy (CAA) burden (IVW *β* = −1.34, 95% CI [−2.39, −0.29], *p* = 0.013) were observed, these did not survive false discovery rate (FDR) correction and therefore should be interpreted with caution (Supporting Information S2: Table 6; Figure 3).

**FIGURE 3:**
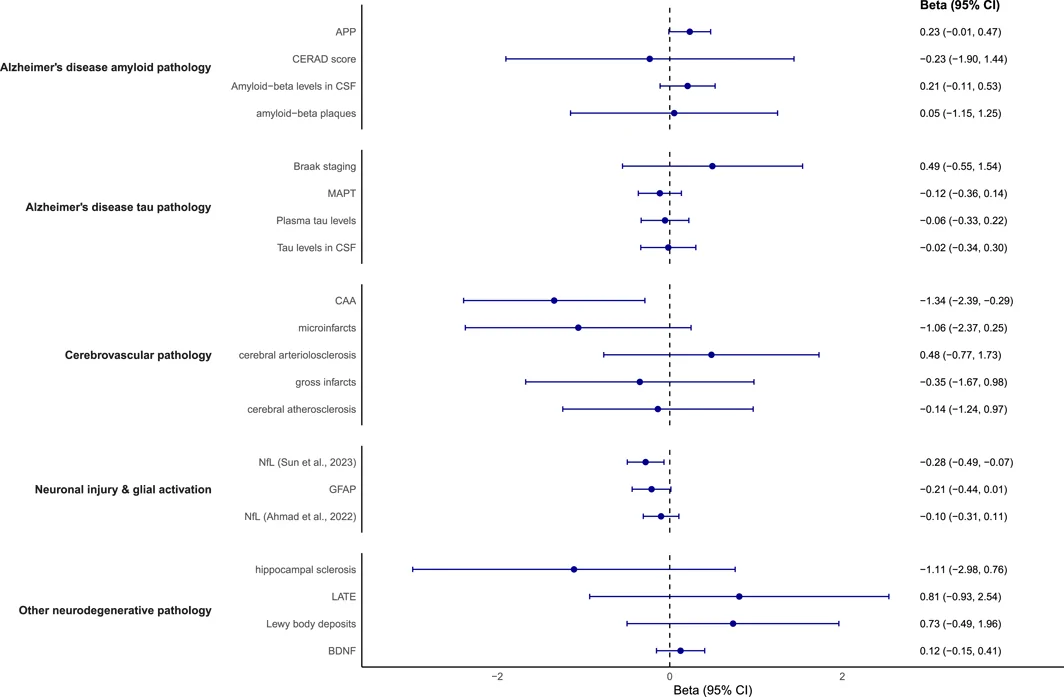
Two‐sample Mendelian randomisation estimates of the effect of genetic liability to opioid use disorder (OUD) on neurobiological and vascular biomarkers. The plot displays beta coefficients and 95% confidence intervals for the associations between genetically predicted OUD and a range of neuropathological and brain‐related phenotypes. Biomarkers are grouped by biological function, including Alzheimer's disease amyloid and tau pathology, neuronal injury and glial activation, other neurodegenerative processes, and cerebrovascular pathology. Source GWAS are detailed in Supporting Information S2: Tables 1 and 2. Estimates were derived using inverse‐variance weighted (IVW) MR. Beta coefficients represent the change in outcome per unit increase in the log‐odds of genetic liability to OUD. Estimates from different scales are displayed on a single axis, so numerical magnitudes are not directly comparable across outcomes.

Further details of the neuroendophenotype analyses are provided in Supporting Information S1: Figure 4.

## 4 Discussion

We used Mendelian randomisation to investigate whether genetic liability to OUD influences inflammatory and neurodegenerative pathways. We found evidence that greater genetically predicted liability to OUD is associated with higher circulating levels of C‐reactive protein (CRP), supporting a link with systemic inflammation. This association was replicated in an independent dataset [10], strengthening the robustness of the finding. We also observed a nominal inverse association between genetic liability to OUD and circulating interleukin‐15 (IL‐15) levels, however, this finding did not survive correction for multiple testing and should be interpreted cautiously.

The absence of colocalisation and the limited number of OUD genetic instruments preclude conclusions regarding shared causal variants or a causal mediating role for CRP. These findings should therefore be considered hypothesis‐generating and require confirmation in larger genetic studies. Beyond CRP, we found little evidence for causal effects of instrumented OUD liability on other outcomes, including Alzheimer's disease–related biomarkers (tau, amyloid‐β), markers of neuroaxonal damage or astroglia activation (NfL, GFAP), or vascular pathology. Nominal associations with NfL and cerebral amyloid angiopathy did not survive correction for multiple testing. These findings should be interpreted in the context of previous literature. While observational studies have reported associations between OUD and dementia risk, the underlying biological mechanisms remain unclear [1, 2]. Our recent MR study provided evidence supporting a causal relationship between OUD and all‐cause dementia [1], but did not find associations with stroke, despite hypotheses implicating cerebrovascular pathways. Together, this suggests mechanisms other than overt vascular disease may be important.

The association with CRP is consistent with clinical studies reporting elevated inflammatory markers in individuals with OUD, which have been linked to cognitive impairment [18]. CRP may be particularly sensitive in this context, reflecting its role as a stable, downstream marker of systemic inflammation, in contrast to more transient cytokines and their relatively underpowered genetic instruments. As a non‐specific marker integrating multiple inflammatory pathways, CRP may capture broader immune dysregulation associated with OUD. CRP GWAS are also substantially larger than those for individual cytokines, providing greater statistical power. This may also suggest that genetic liability to OUD primarily influences broader inflammatory processes rather than specific cytokine pathways. Although IL‐15 also showed a nominally significant association, this finding did not survive multiple testing correction and requires further testing in larger datasets. Several biological mechanisms could underlie this relationship. Chronic opioid exposure is known to activate peripheral and central immune responses, increase pro‐inflammatory signalling, and regularly disrupt systems that include the hypothalamic‐pituitary‐adrenal axis. Opioids may also impair gut barrier function, promoting systemic immune activation. Systemic inflammation, in turn, may influence brain health through effects on the blood–brain barrier and neuroinflammatory pathways, providing a plausible link between OUD, cognitive impairment, and dementia risk [5].

In contrast, we found little evidence linking OUD liability to specific neurodegenerative biomarkers. Previous studies in this area have been inconsistent and often limited by small samples or heterogeneous populations [19]. Our null findings may reflect limited statistical power in available GWAS datasets, particularly for neuropathological markers, and do not exclude more subtle or indirect effects. Larger and more specific datasets, particularly in populations with OUD, will be needed to clarify these relationships.

Key strengths of our study include the use of a causal inference framework such as Mendelian randomisation, which is less susceptible to bias from environmental confounding and reverse causality. We also integrated multiple large‐scale GWAS datasets across biologically relevant inflammatory and neurodegenerative domains. However, several limitations should be noted. Statistical power was limited both for OUD and for some outcomes, particularly neurodegenerative markers. It is also important to note that our exposure represents genetic liability to OUD rather than the clinical phenotype itself. Genetic liability and the clinical disorder may reflect distinct biological mechanisms, and our findings should therefore be interpreted in the context of genetic predisposition rather than manifest disorder. Additionally, several biomarker datasets were derived from indirect plasma protein measurements obtained using different assay platforms, including Olink proteomic and bead‐based immunoassays, which may vary in sensitivity and specificity across proteins and could contribute to measurement heterogeneity. Potential participant overlap between the OUD exposure GWAS and the FinnGen CRP replication dataset could bias two‐sample MR estimates, particularly in the presence of weak instruments; however, all OUD instruments had F‐statistics > 30, reducing the potential for substantial weak‐instrument bias. Nevertheless, this potential overlap should be considered when interpreting the CRP replication findings. Null colocalisation findings across CRP and IL‐15 analyses should also be interpreted cautiously given the assumptions underlying standard coloc models. Residual pleiotropy cannot be excluded, and variation in phenotype definitions across contributing studies may introduce measurement error. Given limited sample sizes in other populations, analyses were restricted to predominantly European ancestry populations, limiting generalizability. Finally, the lack of biomarker data specifically from individuals with OUD may reduce the specificity of our findings.

## 5 Conclusion

Genetic liability to OUD was associated with elevated circulating CRP levels, with this association replicated in an independent dataset, raising the hypothesis that systemic inflammation may be relevant in the relationship between OUD and dementia risk. A nominal association with IL‐15 provides an additional signal that warrants replication. However, we found no evidence for associations with the neurodegenerative or vascular phenotypes examined, although these findings do not exclude Alzheimer's disease or cerebrovascular pathways, particularly given limited power and the use of only three genetic instruments. The inflammatory findings should therefore be considered hypothesis‐generating, particularly given the lack of colocalisation support. Further large‐scale studies, particularly those incorporating well‐characterised OUD populations and longitudinal biomarker data, are needed to clarify the underlying mechanisms of systemic inflammation associated with OUD and inform prevention strategies.

## Ethics Statement

This study used publicly available summary‐level GWAS data from studies with existing ethical approval and participant informed consent.

## Conflicts of Interest

D.G. is the Chief Executive Officer of Sequoia Genetics, a private limited company that works with investors, pharma, biotech, and academia by performing research that leverages genetic data to help inform drug discovery and development. D.G. has financial interests in several biotechnology companies. H.T.C is employed by Sequoia Genetics. SBu is an employee of Sequoia Genetics, a private limited company that works with investors, pharma, biotech, and academia by performing research that leverages genetic data to help inform drug discovery and development. Dr. Kranzler is a member of advisory boards for Altimmune and Clearmind Medicine; a consultant to Sobrera Pharmaceuticals, Altimmune, Lilly, Ribocure, and Boehringer‐Ingelheim; and the recipient of research funding and medication supplies for an investigator‐initiated study from Alkermes and company‐initiated studies by Altimmune and Lilly.

## Supporting information


Supporting Information S1

Supporting Information S2

## Data Availability

Summary statistics from multiple genome‐wide association studies were obtained from publicly available datasets, with additional summary statistics provided through collaboration or direct author correspondence. Details of all datasets included in the analyses are provided in Supporting Information S2: Tables 1 and 2. The analysis code used in this study is available upon reasonable request. Further information regarding the datasets can be provided if required.
