# Supplementary material for: Genetic Liability to Opioid Use Disorder and Dementia‐Related Biological Pathways: A Mendelian Randomisation Study
Source: Int J Geriatr Psychiatry. 2026 Nov 6;41(11):e70270. doi: 10.1002/gps.70270 (PMC13743915; doi:10.1002/gps.70270)
Supplement: Supplementary file 1 [file GPS-41-e70270-s001.docx]

# Supplementary Figures Index

[Supplementary Figures Index	1](#_Toc240466626)

[Supplementary Figure 1.	3](#_Toc240466627)

[Bayesian colocalization analyses between genetic liability to opioid use disorder and circulating C-reactive protein levels in the Kurki et al. dataset at the OPRM1 and FURIN loci.	3](#_Toc240466628)

[Supplementary Figure 2.	4](#_Toc240466629)

[Bayesian colocalization analyses between genetic liability to opioid use disorder and circulating interleukin-15 levels at the OPRM1 and FURIN loci.	4](#_Toc240466630)

[Supplementary Figure 3.	5](#_Toc240466631)

[Mendelian randomisation estimates for associations between genetic liability to opioid use disorder and circulating inflammatory markers.	5](#_Toc240466632)

[1. OUD–CRP (Said et al., 2022)	5](#_Toc240466633)

[2. OUD–CRP (Kurki et al., 2023)	5](#_Toc240466634)

[3. OUD–IL-15	5](#_Toc240466635)

[4. OUD–IL-4	6](#_Toc240466636)

[5. OUD–IL-1β	6](#_Toc240466637)

[6. OUD–IL-18	6](#_Toc240466638)

[7. OUD–IL-6	7](#_Toc240466639)

[8. OUD–IL-1α	7](#_Toc240466640)

[9. OUD–MCP-1 (CCL2)	7](#_Toc240466641)

[Supplementary Figure 4.	8](#_Toc240466642)

[Mendelian randomisation estimates for associations between genetic liability to opioid use disorder and dementia-related neuroendophenotypes.	8](#_Toc240466643)

[1.	OUD–NfL (Ahmad et al., 2022; bead-based immunoassay)	8](#_Toc240466644)

[2. OUD–NfL (Sun et al., 2023; Olink proteomic assay)	8](#_Toc240466645)

[3. OUD–GFAP	9](#_Toc240466646)

[4. OUD–APP	9](#_Toc240466647)

[5. OUD–MAPT	9](#_Toc240466648)

[6. OUD–BDNF	10](#_Toc240466649)

[7. OUD–plasma tau	10](#_Toc240466650)

[8. OUD–CSF tau	10](#_Toc240466651)

[9. OUD–CSF amyloid-β	11](#_Toc240466652)

[10. OUD–cerebral amyloid angiopathy (CAA)	11](#_Toc240466653)

[11. OUD–arteriolosclerosis	11](#_Toc240466654)

[12. OUD–atherosclerosis	12](#_Toc240466655)

[13. OUD–CERAD score	12](#_Toc240466656)

[14. OUD–amyloid-β plaques	12](#_Toc240466657)

[15. OUD–Braak stage	13](#_Toc240466658)

[16. OUD–gross infarcts	13](#_Toc240466659)

[17. OUD–hippocampal sclerosis	13](#_Toc240466660)

[18. OUD–LATE	14](#_Toc240466661)

[19. OUD–Lewy bodies	14](#_Toc240466662)

[20. OUD–microinfarcts	14](#_Toc240466663)

# Supplementary Figure 1.

# Bayesian colocalization analyses between genetic liability to opioid use disorder and circulating C-reactive protein levels in the Kurki et al. dataset at the *OPRM1* and *FURIN* loci.

Supplementary F1. Bayesian colocalization analyses examining shared causal variants between genetic liability to opioid use disorder (OUD) and circulating C-reactive protein (CRP) levels in the independent Kurki et al. dataset at the *OPRM1* and *FURIN* loci. Posterior probabilities supported association with OUD liability alone (PP.H1), with limited evidence for colocalization between OUD and CRP signals under the single-causal-variant coloc model (all PP.H4 < 0.01).

# Supplementary Figure 2.

# Bayesian colocalization analyses between genetic liability to opioid use disorder and circulating interleukin-15 levels at the *OPRM1* and *FURIN* loci.

Supplementary F2. Bayesian colocalization analyses examining shared causal variants between genetic liability to opioid use disorder (OUD) and circulating interleukin-15 (IL-15) levels at the *OPRM1* and *FURIN l*oci. Posterior probabilities supported association with OUD liability alone (PP.H1), with limited evidence for colocalization between OUD and IL-15 signals under the single-causal-variant coloc model (all PP.H4 < 0.02).

# Supplementary Figure 3.

# **Mendelian randomisation estimates for associations between genetic liability to opioid use disorder and circulating inflammatory markers.**

## 1. OUD–CRP (Said et al., 2022)

## 2. OUD–CRP (Kurki et al., 2023)

## 3. OUD–IL-15

## 4. OUD–IL-4

## 5. OUD–IL-1β

## 6. OUD–IL-18

## 7. OUD–IL-6

## 8. OUD–IL-1α

## 9. OUD–MCP-1 (CCL2)

**Supplementary Figure 3.** Mendelian randomisation analyses examining associations between genetic liability to opioid use disorder (OUD) and circulating inflammatory markers. For each outcome, the scatterplot shows SNP–OUD and SNP–outcome associations together with estimates obtained using inverse-variance weighted, MR-Egger, weighted median, simple mode, and weighted mode methods. The corresponding forest plot shows SNP-specific Wald ratio estimates together with the overall inverse-variance weighted and MR-Egger estimates. CRP was evaluated separately using the Said et al. (2022) and Kurki et al. (2023) datasets. CRP, C-reactive protein; IL, interleukin; MCP-1, monocyte chemoattractant protein-1; CCL2, C-C motif chemokine ligand 2.

# Supplementary Figure 4.

# **Mendelian randomisation estimates for associations between genetic liability to opioid use disorder and dementia-related neuroendophenotypes.**

## 1. OUD–NfL (Ahmad et al., 2022; bead-based immunoassay)

## 2. OUD–NfL (Sun et al., 2023; Olink proteomic assay)

## 3. OUD–GFAP

## 4. OUD–APP

## 5. OUD–MAPT

## 6. OUD–BDNF

## 7. OUD–plasma tau

## 8. OUD–CSF tau

## 9. OUD–CSF amyloid-β

## 10. OUD–cerebral amyloid angiopathy (CAA)

## 11. OUD–arteriolosclerosis

## 12. OUD–atherosclerosis

## 13. OUD–CERAD score

## 14. OUD–amyloid-β plaques

## 15. OUD–Braak stage

## 16. OUD–gross infarcts

## 17. OUD–hippocampal sclerosis

## 18. OUD–LATE

## 19. OUD–Lewy bodies

## 20. OUD–microinfarcts

**Supplementary Figure 4.** Mendelian randomisation analyses examining associations between genetic liability to opioid use disorder (OUD) and dementia-related neuroendophenotypes. For each outcome, the scatterplot shows SNP–OUD and SNP–outcome associations together with estimates obtained using inverse-variance weighted, MR-Egger, weighted median, simple mode, and weighted mode methods. The corresponding forest plot shows SNP-specific Wald ratio estimates together with the overall inverse-variance weighted and MR-Egger estimates. Neurofilament light chain (NfL) was assessed using two independent datasets employing different measurement platforms. NfL, neurofilament light chain; GFAP, glial fibrillary acidic protein; APP, amyloid precursor protein; MAPT, microtubule-associated protein tau; BDNF, brain-derived neurotrophic factor; CSF, cerebrospinal fluid; CAA, cerebral amyloid angiopathy; CERAD, Consortium to Establish a Registry for Alzheimer’s Disease; LATE, limbic-predominant age-related TDP-43 encephalopathy.
